# Orthohantavirus Infection Mimicking Acute Viral Hepatitis: An Underrecognized Clinical Presentation

**DOI:** 10.3390/pathogens15060632

**Published:** 2026-06-15

**Authors:** Francesco De Maria, Francesco Branda, Giancarlo Ceccarelli, Fabio Scarpa, Massimo Ciccozzi, Alessandro Russo

**Affiliations:** 1 Infectious and Tropical Diseases Unit, “Renato Dulbecco” Teaching Hospital of Catanzaro, 88100 Catanzaro, Italy; francescodemaria16@gmail.com (F.D.M.); a.russo@unicz.it (A.R.); 2 Unit of Medical Statistics and Molecular Epidemiology, Università Campus Bio-Medico di Roma, 00128 Rome, Italy; m.ciccozzi@unicampus.it; 3 Department of Public Health and Infectious Diseases, University Hospital Policlinico Umberto I, Sapienza University of Rome, 00185 Rome, Italy; giancarlo.ceccarelli@uniroma1.it; 4 Department of Biomedical Sciences, University of Sassari, 07100 Sassari, Italy; fscarpa@uniss.it

**Keywords:** orthohantavirus, hantavirus, acute viral hepatitis, hypertransaminasemia, endothelial dysfunction, thrombocytopenia, differential diagnosis, zoonosis

## Abstract

Orthohantavirus infections are classically associated with hemorrhagic fever with renal syndrome (HFRS) in Eurasia and hantavirus cardiopulmonary syndrome (HCPS) in the Americas. However, accumulating evidence indicates that the clinical spectrum is considerably broader, with frequent involvement of organ systems beyond the kidney and lung. Hepatic manifestations, in particular, may mimic acute viral hepatitis, leading to diagnostic challenges and underrecognition. This paper synthesizes published evidence on hepatic involvement in orthohantavirus infection, with a focus on clinical presentation, pathogenic mechanisms, differential diagnosis, biomarkers, and public health implications. Relevant literature was identified through searches of peer-reviewed articles, with emphasis on studies reporting hypertransaminasemia, hepatitis-like illness, and liver injury in confirmed hantavirus infections. Mild to moderate elevations in aminotransferases are common during acute orthohantavirus infection, and in some patients the clinical picture may be dominated by fever, thrombocytopenia, and hepatitis-like abnormalities, closely resembling dengue, leptospirosis, or classical viral hepatitis. Hepatic injury appears to result primarily from systemic endothelial dysfunction, immune-mediated inflammation, and microvascular leakage rather than direct hepatocytopathic effects. Emerging biomarkers of severity, including thrombocytopenia, neutrophil-to-lymphocyte ratio, soluble thrombomodulin, and IL-6 trans-signaling, reflect widespread vascular and inflammatory activation. Diagnostic delays are frequent, particularly in non-endemic regions, due to low clinical awareness and overlapping features with more common febrile hepatotropic syndromes. Orthohantavirus infection should be considered in the differential diagnosis of acute febrile illness with unexplained hypertransaminasemia and thrombocytopenia, especially when epidemiological clues suggest rodent exposure or compatible environmental contexts. Recognizing hepatic involvement as part of a systemic endothelial syndrome may improve diagnostic accuracy, reduce underreporting, and facilitate earlier supportive management. Increased awareness among hepatologists, infectious disease specialists, and emergency physicians is warranted.

## 1. Introduction

Orthohantaviruses are enveloped, negative-sense RNA viruses belonging to the family *Hantaviridae*, order *Bunyavirales*, and represent a genetically and ecologically diverse group of zoonotic pathogens transmitted to humans primarily through inhalation of aerosols contaminated with excreta from infected rodent reservoir hosts [1,2,3]. More than 40 viral species have been identified to date, of which approximately a dozen are recognized as pathogenic in humans. These species differ substantially in their geographic distribution, reservoir hosts, clinical phenotype, and virulence. The principal clinically relevant orthohantaviruses, together with their reservoirs, geographic range, associated syndromes, and approximate case fatality rates, are summarised in Table 1.

However, over the past ten to fifteen years, a growing body of evidence has challenged this binary classification, suggesting that the clinical spectrum of orthohantavirus disease is considerably broader and more heterogeneous than initially recognized. Beyond the classical renal and pulmonary manifestations, these infections may involve multiple organ systems through mechanisms largely driven by endothelial dysfunction, immune activation, and microvascular leakage [1,3,27].

The vascular endothelium represents a central target of orthohantavirus infection. Viral tropism for endothelial cells contributes to increased capillary permeability, dysregulated inflammatory signaling, and widespread tissue involvement without overt cytopathic destruction [1,28]. This distinctive feature, i.e., functional impairment without massive cell lysis, is fundamental to understanding why orthohantavirus infections can present with diverse and overlapping clinical pictures, often mimicking other systemic febrile illnesses. This endothelial-centered pathogenesis likely explains the heterogeneity of clinical manifestations reported across different hantavirus species, viral genotypes, and geographic settings. Recent experimental studies have further highlighted the role of pericyte infection, innate immune evasion, and species-specific host responses in shaping disease severity and organ involvement [29,30,31,32,33,34,35]. For instance, Andes virus infection of pericytes has been shown to enhance endothelial permeability, suggesting that the microvascular unit as a whole, not just endothelial cells, contributes to disease pathogenesis [29].

Although renal impairment (ranging from mild proteinuria to acute kidney injury requiring dialysis) and pulmonary edema (ranging from mild interstitial infiltrates to fulminant respiratory failure) remain the most recognized hallmarks of severe disease, hepatic abnormalities are increasingly being reported in both endemic and non-endemic settings. Mild to moderate elevations in aminotransferases, particularly aspartate aminotransferase (AST) and alanine aminotransferase (ALT), are not uncommon during acute infection, and in some patients the clinical presentation may resemble acute viral hepatitis or other febrile hepatotropic syndromes [3,36,37]. In a subset of cases, hypertransaminasemia may be accompanied by jaundice, right upper quadrant discomfort, hepatomegaly, or even mild coagulopathy, further blurring the distinction between hantavirus disease and primary hepatitis. This aspect remains underappreciated in routine clinical practice, particularly outside reference centers familiar with hantavirus epidemiology.

The possibility of hepatic involvement is clinically relevant for several interconnected reasons. First, patients presenting with fever, hypertransaminasemia, thrombocytopenia, and nonspecific systemic symptoms (such as myalgia, headache, nausea, vomiting, and abdominal pain) are frequently investigated for dengue, leptospirosis, malaria, typhoid fever, or classical hepatotropic viruses (hepatitis A, B, C, E, Epstein–Barr virus, cytomegalovirus) before orthohantavirus infection is considered [36,38,39]. This diagnostic tunnel vision can lead to unnecessary testing, delayed supportive care, and missed opportunities for early monitoring of complications such as capillary leakage, shock, or acute kidney injury. Second, the expanding geographic distribution of rodent reservoirs, climate-associated ecological changes, deforestation, agricultural intensification, and increasing international mobility may contribute to diagnostic delays in areas traditionally considered non-endemic or low-incidence [40,41]. Even in Europe, where several hantavirus species (e.g., Puumala, Dobrava, Seoul, Tula) circulate, underdiagnosis is common, and seroprevalence studies often reveal higher exposure rates than clinically reported cases. Third, growing recognition of atypical and severe systemic phenotypes suggests that liver involvement may reflect broader endothelial and immune dysregulation rather than isolated hepatocellular injury [1,3,42]. In this context, the degree of hypertransaminasemia might serve as a surrogate marker of systemic inflammatory burden and vascular dysfunction, potentially correlating with disease severity and prognosis.

Several recent studies have also explored biomarkers associated with disease severity, including inflammatory cytokines (such as IL-6, TNF-α, and IL-10), neutrophil-to-lymphocyte ratio (NLR), coagulation abnormalities (prolonged prothrombin time, elevated D-dimer, thrombocytopenia), and markers of endothelial activation (soluble thrombomodulin, vascular cell adhesion molecule-1, angiopoietin-2) [43,44,45,46,47]. These findings reinforce the concept of orthohantavirus infection as a systemic inflammatory vascular disease with heterogeneous organ involvement, where the pattern of organ dysfunction depends on a complex interplay between viral strain, host genetic background, immune response, and possibly environmental factors. In parallel, advances in experimental models, including human endothelial cell cultures, pericyte co-culture systems, and small animal models, have improved understanding of viral-host interactions, including mechanisms of immune escape (e.g., interference with interferon signaling), endothelial barrier disruption (e.g., Vascular Endothelial Growth Factor (VEGF) sensitization, Src kinase activation), and extrapulmonary manifestations [30,31,32,33,34,35].

Importantly, the recognition of hepatic involvement is not merely an academic exercise. From a practical standpoint, identifying orthohantavirus infection in a patient presenting with a hepatitis-like syndrome can prevent invasive diagnostic procedures (such as liver biopsy), avoid unnecessary antiviral or immunosuppressive therapies, and guide appropriate supportive management, including fluid balance monitoring, avoidance of nephrotoxic drugs, early recognition of capillary leakage, and timely referral to specialized care if renal or respiratory complications ensue. Furthermore, accurate diagnosis has public health implications, including case reporting, epidemiological surveillance, and, in the case of Andes virus, contact tracing due to documented person-to-person transmission [48].

In this work, we discuss the emerging evidence supporting hepatic involvement during orthohantavirus infection, with particular attention to hepatitis-like presentations, underlying pathogenic mechanisms, differential diagnostic challenges, and potential clinical implications. We also highlight the need for increased awareness among infectious disease specialists, hepatologists, emergency physicians, and intensivists, particularly when evaluating patients with acute febrile illness and unexplained liver enzyme abnormalities, especially in the presence of thrombocytopenia or epidemiological clues suggesting rodent exposure. Specifically, we first examine the expanding multisystem spectrum of orthohantavirus disease, with emphasis on its nature as a systemic endothelial disorder (Section 2). We then focus on hepatic involvement, its clinical manifestations, proposed pathogenic mechanisms, and immunopathogenesis (Section 3), before addressing the differential diagnostic challenges posed by overlap with dengue, leptospirosis, and classical viral hepatitis (Section 4). Biomarkers of severity and prognostic models (Section 5), as well as public health implications including ecological drivers and underreporting (Section 7), are discussed in the concluding sections (Section 8).

## 2. Expanding Clinical Spectrum Beyond Renal and Pulmonary Disease

Orthohantavirus infections have historically been classified according to their predominant clinical phenotype, namely hemorrhagic fever with renal syndrome in Eurasia and hantavirus cardiopulmonary syndrome in the Americas [1,2,3,27]. This binary classification has long served as a useful framework for clinicians, guiding diagnostic algorithms, clinical management decisions, and public health surveillance strategies. However, accumulating evidence over the past two decades has increasingly challenged the notion that orthohantavirus disease can be adequately described by a strict kidney-versus-lung dichotomy. A growing body of clinical, epidemiological, and experimental data suggests that these infections are more accurately understood as systemic endothelial disorders, characterized by heterogeneous organ involvement, markedly variable inflammatory responses, and a clinical spectrum that frequently extends well beyond the renal and pulmonary systems [1,3].

### 2.1. Endothelial Dysfunction and Immune Activation as Drivers of Systemic Disease

A defining feature of orthohantavirus pathogenesis is the ability of the virus to infect endothelial cells without inducing extensive direct cytopathic damage [1,49]. Unlike classical cytopathic viruses that cause overt cell lysis and tissue destruction, orthohantaviruses establish a productive infection within endothelial cells while largely preserving their structural integrity. This paradoxical finding, infection without destruction, has shifted the paradigm toward understanding hantavirus disease as a functional vasculopathy rather than a primary cytolytic illness. Disease severity does not appear to correlate simply with viral load or cytopathic effect, but rather results from a complex and dynamic interaction between viral replication, endothelial dysfunction, immune activation, and dysregulated vascular permeability. The consequent capillary leakage syndrome represents the central pathophysiological event, contributing directly to tissue edema, hemodynamic instability, thrombocytopenia, and the multiorgan manifestations observed during severe disease [1,3,27,28].

**Figure 1:**
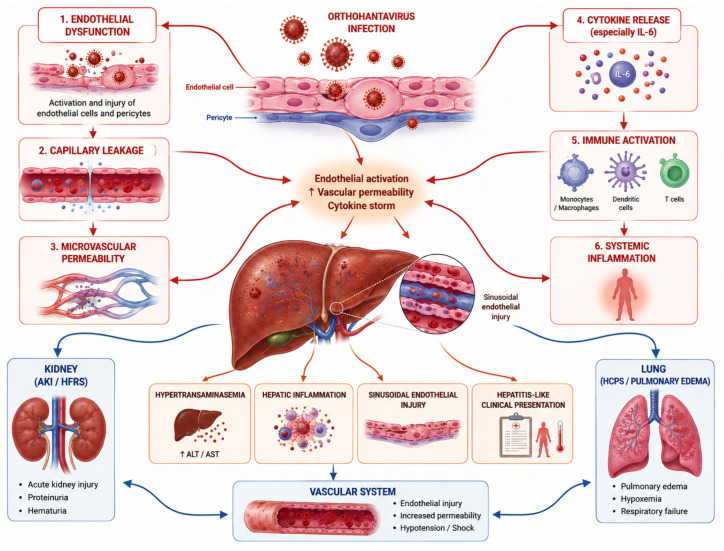
Pathogenic cascade and systemic clinical manifestations of orthohantavirus infection. Red arrows indicate the progression of endothelial injury and vascular dysfunction (steps 1–3), leading to organ-specific effects. Blue arrows indicate immune-mediated amplification pathways (steps 4–6), including cytokine release and systemic inflammation. The figure illustrates the sequence of events linking orthohantavirus infection to multiorgan disease. Infection of endothelial cells and pericytes triggers endothelial activation, increased vascular permeability, and capillary leakage. In parallel, release of pro-inflammatory cytokines, particularly IL-6, from infected endothelial cells and activated immune cells (monocytes, macrophages, dendritic cells, T cells) amplifies immune activation and systemic inflammation. This cascade results in renal involvement (AKI, proteinuria, hematuria), pulmonary involvement (edema, hypoxemia, respiratory failure), hepatic involvement (hypertransaminasemia, sinusoidal endothelial injury), and hemodynamic instability (hypotension, shock, tissue hypoxia).

The pathogenic cascade that underlies this systemic involvement is illustrated in Figure 1. The process begins with infection of endothelial cells and, as recently recognized, of pericytes, the contractile cells that envelop capillary endothelium and regulate microvascular stability. This infection leads to activation and injury of the microvascular unit, triggering increased vascular permeability and microvascular leakage. In parallel, infected endothelial cells and activated immune cells release a cascade of pro-inflammatory cytokines, most notably interleukin-6, which further amplifies endothelial activation and creates a self-sustaining loop of inflammation and vascular dysfunction. This cytokine storm ultimately drives systemic inflammation and accounts for the diverse organ manifestations that characterize severe orthohantavirus disease. The cellular and molecular mechanisms underlying endothelial barrier disruption, pericyte involvement, innate immune evasion, and cytokine-mediated injury are discussed in detail in Section 6.

### 2.2. Clinical Heterogeneity, Atypical Presentations, and Changing Epidemiology

This systemic inflammatory profile likely contributes to the increasingly recognized spectrum of extrapulmonary and extraprenal manifestations. Neurological involvement, ranging from headache and aseptic meningitis to encephalitis and seizures, has been described. Gastrointestinal symptoms, including nausea, vomiting, abdominal pain, and diarrhea, are common and often precede more specific organ dysfunction. Hepatic abnormalities, ranging from mild hypertransaminasemia to clinically overt hepatitis-like presentations, are increasingly reported. Coagulation disorders, including thrombocytopenia and disseminated intravascular coagulation, frequently accompany severe disease. Finally, severe multiorgan failure involving the renal, pulmonary, hepatic, and cardiovascular systems has been documented in both Old World and New World hantavirus infections [2,3,27,50]. In a substantial subset of patients, the initial clinical picture may be dominated by nonspecific febrile illness associated with thrombocytopenia and elevated liver enzymes, without early evidence of renal or pulmonary involvement. This presentation can delay diagnostic suspicion and lead to initial misclassification as dengue, leptospirosis, or acute viral hepatitis, with important implications for patient management and public health reporting.

The clinical heterogeneity of orthohantavirus infection has become particularly evident during outbreaks caused by Andes virus in South America. Unlike most other orthohantaviruses, which are transmitted exclusively from rodents to humans, Andes virus has demonstrated the capacity for person-to-person transmission, including transmission chains associated with so-called super-spreader events [48]. These outbreaks have highlighted not only the epidemic potential of specific orthohantavirus species but also the broad variability in disease severity that can occur even within a single outbreak. Clinical presentations have ranged from mild systemic illness, sometimes with only transient fever and mild transaminase elevation, to fulminant cardiopulmonary collapse and multiorgan dysfunction requiring intensive care support, extracorporeal membrane oxygenation, or renal replacement therapy [3,48,51].

ANDV deserves particular attention given its unique epidemiological profile among orthohantaviruses. It circulates primarily in the southern cone of South America, with the highest burden of disease reported in Argentina and Chile, and additional cases documented in Bolivia, Uruguay, and Paraguay [3,50]. Its principal rodent reservoir is the long-tailed pygmy rice rat (*Oligoryzomys longicaudatus*), a small cricetid rodent inhabiting shrubland and forest-edge environments at various altitudes. Unlike all other known pathogenic orthohantaviruses, which are transmitted exclusively through rodent-to-human contact, Andes virus has demonstrated the capacity for sustained person-to-person transmission, a feature with profound implications for outbreak dynamics and infection control [50]. Transmission has been documented through close and prolonged contact with symptomatic patients, most notably in household and healthcare settings, and has given rise to so-called super-spreader events in which a single index case generated multiple secondary infections [50]. The clinical syndrome caused by Andes virus is a severe form of HCPS, with case fatality rates reported between 35% and 40% in most series. Disease onset is typically preceded by a febrile prodrome lasting several days, after which rapid cardiopulmonary deterioration may ensue, often requiring mechanical ventilation and haemodynamic support. The combination of high lethality, person-to-person transmissibility, and geographic expansion into peri-urban areas makes Andes virus one of the most epidemiologically significant orthohantavirus species currently recognized.

Recent epidemiological studies further suggest that climate change, ecological disruption, and expanding rodent-human interactions may influence both viral circulation and human exposure risk in ways that are only beginning to be understood [40]. Warmer temperatures, altered precipitation patterns, habitat fragmentation, and agricultural intensification can all affect rodent population dynamics, food availability, and the frequency and intensity of human–rodent contact. Consequently, clinicians practicing outside traditionally endemic regions may increasingly encounter atypical presentations of orthohantavirus infection, including cases initially interpreted as acute hepatitis, dengue-like illness, leptospirosis, or sepsis of unclear origin.

The central role of endothelial dysfunction and systemic inflammation in driving these diverse manifestations is illustrated in Figure 1, which depicts the cascade from endothelial and pericyte infection to cytokine release, immune activation, and ultimately multiorgan involvement.

The expanding multisystem spectrum of orthohantavirus infection is summarized in Table 2, which provides an overview of the main clinical domains involved, their characteristic manifestations, the proposed pathogenic mechanisms, and key references.

## 3. Hepatic Involvement in Orthohantavirus Infection

Although hepatic manifestations are not traditionally considered among the defining features of orthohantavirus disease, abnormalities in liver function tests have been repeatedly documented in patients with both hemorrhagic fever with renal syndrome and hantavirus cardiopulmonary syndrome [3,27,36]. In most cases, liver involvement appears mild to moderate in severity and is characterized by transient elevations in aminotransferases, particularly aspartate aminotransferase and alanine aminotransferase. However, accumulating evidence suggests that hepatic abnormalities may occasionally represent a clinically relevant component of disease presentation rather than an incidental laboratory finding. Understanding the full spectrum of hepatic involvement is essential for improving diagnostic accuracy and avoiding misclassification, especially in patients presenting with fever and jaundice or unexplained hypertransaminasemia.

### 3.1. Clinical Evidence of Hepatic Involvement

Several studies have described hepatitis-like syndromes occurring during acute orthohantavirus infection. Historical observations from Europe and Asia reported elevated liver enzymes in patients with confirmed hantavirus infection, sometimes associated with jaundice, systemic inflammatory symptoms, and diagnostic suspicion of acute viral hepatitis [36,37]. In a study conducted in Spain, Lledó and colleagues identified serological evidence of hantavirus infection among patients presenting with pneumonia, renal disease, and hepatitis, suggesting that hepatic involvement may be more frequent than previously recognized [37]. Similar observations were reported in patients with unexplained hypertransaminasemia, further supporting the possibility that orthohantavirus infection may occasionally enter the differential diagnosis of acute hepatitis syndromes [36].

The mechanisms underlying hepatic injury remain incompletely understood. Nevertheless, the systemic endothelial dysfunction that characterizes orthohantavirus infection likely contributes substantially to liver involvement. The hepatic microcirculation is particularly vulnerable to inflammatory and vascular perturbations, and increased endothelial permeability may promote sinusoidal dysfunction, tissue edema, and secondary hepatocellular injury [1,3]. Unlike classical hepatotropic viruses such as hepatitis A, B, C, or E, however, current evidence does not support a predominant direct cytopathic effect within hepatocytes. Instead, liver abnormalities appear to reflect broader systemic inflammatory and vascular processes that secondarily affect the liver.

Clinical overlap with other tropical and febrile illnesses may further obscure recognition of hepatic manifestations associated with orthohantavirus infection. Patients frequently present with fever, malaise, thrombocytopenia, elevated transaminases, and gastrointestinal symptoms, a constellation that may initially suggest dengue, leptospirosis, or acute viral hepatitis rather than hantavirus disease [36,38,39]. This diagnostic challenge may be particularly relevant in areas where clinicians are less familiar with orthohantavirus epidemiology, as well as in non-endemic regions where the disease is not routinely considered.

Recent studies have reinforced this concern, as summarized in Table 3. Ekanayake and colleagues identified probable hantavirus infections among patients initially evaluated for suspected dengue infection in Sri Lanka, highlighting the potential for clinical misclassification in both endemic and emerging settings [39]. Similarly, overlap between hantavirus infection and leptospirosis has been increasingly recognized due to shared clinical and laboratory features, including thrombocytopenia, acute kidney injury, inflammatory activation, and hepatic abnormalities [38]. In both scenarios, the presence of hypertransaminasemia may misdirect diagnostic efforts toward more common hepatotropic or tropical infections, delaying appropriate management.

The degree of hepatic involvement may also correlate with overall disease severity. Severe systemic phenotypes characterized by extensive endothelial injury, coagulation abnormalities, and cytokine dysregulation may present with more pronounced liver dysfunction as part of a broader multiorgan process [3,42,50]. In critically ill patients, hepatic abnormalities may therefore represent an indirect marker of systemic inflammatory burden and vascular dysfunction rather than isolated hepatic disease. Conversely, mild hypertransaminasemia may occur even in the absence of overt renal or pulmonary failure, suggesting that liver enzyme elevation can be an early or even dominant feature in some patients.

Importantly, the available literature likely underestimates the true prevalence of hepatic involvement during orthohantavirus infection. Many studies primarily focus on renal or cardiopulmonary outcomes, while liver enzyme abnormalities are often reported only as secondary findings or are not systematically collected. In addition, mild elevations in transaminases may remain clinically overlooked in the context of severe renal failure or respiratory compromise, where clinical attention is naturally directed toward life-threatening organ dysfunction. Consequently, the hepatotropic potential of orthohantavirus infection remains insufficiently characterized and probably underrecognized in routine clinical practice, particularly outside specialized reference centers.

### 3.2. Mechanisms of Hepatic Injury

The mechanisms responsible for hepatic involvement during orthohantavirus infection are likely multifactorial and remain only partially understood. Current evidence suggests that liver abnormalities primarily reflect systemic endothelial dysfunction, immune-mediated inflammation, and microvascular injury rather than direct hepatocellular viral cytotoxicity [1,3,28]. This conceptual framework is essential for distinguishing orthohantavirus-associated hepatitis from classical viral hepatitis and for guiding appropriate clinical management.

A central pathogenic feature of orthohantavirus infection is the disruption of endothelial barrier integrity. Following infection, endothelial cells undergo profound functional alterations characterized by increased vascular permeability, dysregulated inflammatory signaling, and enhanced sensitivity to permeability mediators such as vascular endothelial growth factor [1,28]. Gavrilovskaya and colleagues demonstrated that hantaviruses sensitize endothelial cells to VEGF-induced hyperpermeability, while angiopoietin-1 and sphingosine-1-phosphate may exert protective effects on endothelial stability [28]. These findings support the concept that capillary leakage is a core mechanism underlying systemic organ involvement, including the liver.

Within the liver, endothelial dysfunction may specifically affect the sinusoidal microvasculature, leading to altered hepatic perfusion, inflammatory cell recruitment, and secondary hepatocellular stress. The hepatic sinusoidal network is highly specialized and tightly regulated, with fenestrated endothelial cells that facilitate the exchange of nutrients, waste products, and immune cells between the blood and hepatocytes. Even modest disturbances in sinusoidal vascular permeability may therefore contribute to biochemical evidence of liver injury. In this context, elevated aminotransferases may represent the hepatic expression of a broader endothelial inflammatory syndrome rather than isolated viral hepatitis.

Immune activation appears to play an equally important role in hepatic injury. Severe orthohantavirus disease is characterized by robust cytokine responses involving IL-6, tumor necrosis factor-alpha, interferon signaling pathways, and multiple other inflammatory mediators associated with vascular dysfunction [31,32,42]. Recent work by Maleki and colleagues demonstrated that IL-6 trans-signaling contributes directly to endothelial barrier dysfunction in hantavirus-infected cells and correlates with disease severity in HFRS patients [42]. These findings are particularly relevant because excessive cytokine activation may amplify hepatic inflammation indirectly through systemic immune dysregulation, even in the absence of direct viral infection of hepatocytes.

Innate immune responses and antiviral signaling pathways further contribute to disease heterogeneity. Orthohantaviruses have evolved several mechanisms capable of modulating host innate immunity, thereby influencing endothelial activation and inflammatory responses [30,31,32,35]. Viral interference with interferon pathways, altered cytokine production, and host-specific immune interactions may partly explain why some patients develop mild self-limited illness with only minimal transaminase elevation, whereas others progress to severe systemic disease with marked hepatic involvement and multiorgan failure [30,31,32,35].

Recent experimental studies have also highlighted the importance of non-endothelial vascular cells in disease pathogenesis. Infection of pericytes by Andes virus has been shown to enhance endothelial permeability and inflammatory dysregulation, suggesting that vascular barrier dysfunction involves coordinated alterations across multiple cellular compartments [29]. Within the liver, pericyte-like cells known as hepatic stellate cells may play analogous roles in sinusoidal regulation, although this has not yet been directly investigated in the context of hantavirus infection. Nevertheless, this observation further reinforces the concept of orthohantavirus infection as a diffuse vascular inflammatory disorder with secondary organ manifestations.

Differences between Old World and New World hantaviruses may additionally influence patterns of organ involvement, including the liver. Jeyachandran and colleagues recently demonstrated distinct cellular tropisms between hantavirus species, with divergent interactions involving respiratory epithelial cells, endothelial cells, and immune pathways [44]. Such biological variability may partly account for differences in clinical phenotype, disease severity, and extrapulmonary manifestations observed across geographic regions, although systematic comparisons of hepatic involvement between viral species remain limited.

Coagulation abnormalities likely represent another relevant contributor to hepatic injury. Thrombocytopenia, endothelial activation, platelet dysfunction, and altered coagulation pathways are common findings during acute infection [3,43,46]. In severe cases, microvascular injury and systemic inflammatory coagulopathy may exacerbate tissue hypoperfusion and organ dysfunction, including within the liver. Emerging biomarkers such as soluble thrombomodulin further support the role of endothelial damage in severe disease phenotypes [47]. The liver, as a highly vascular organ with a low flow sinusoidal system, may be particularly susceptible to these microvascular insults.

**Figure 2:**
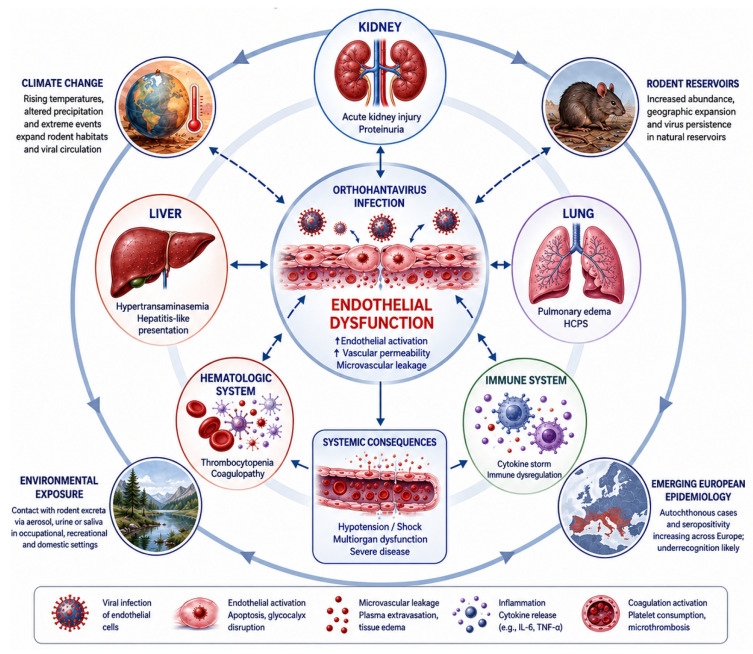
Integrated pathogenetic pathways and clinical manifestations of orthohantavirus infection. Solid arrows denote direct pathogenic cascades (endothelial infection → activation → microvascular leakage → organ injury). Dashed arrows indicate indirect amplification pathways (cytokine release, coagulation activation, immune dysregulation) and contextual factors (climate change, emerging European epidemiology). The diagram illustrates the cascade from environmental exposure to rodent excreta through orthohantavirus infection of endothelial cells, leading to endothelial activation, glycocalyx disruption, and microvascular leakage. This process triggers cytokine release (IL-6, TNF-α), coagulation activation with platelet consumption, and systemic inflammation. Endothelial dysfunction serves as the central pathogenic hub, driving multiorgan involvement including acute kidney injury, pulmonary edema/HCPS, hypertransaminasemia with hepatitis-like presentation, and thrombocytopenia/coagulopathy. Systemic consequences include hypotension, shock, and multiorgan dysfunction. The diagram also highlights the role of climate change in expanding rodent habitats and viral circulation, as well as emerging European epidemiology with increasing autochthonous cases and underrecognition.

Overall, available evidence suggests that hepatic involvement during orthohantavirus infection should not be interpreted as classical hepatotropic viral injury. Instead, it appears to represent a secondary manifestation of widespread endothelial dysfunction, immune activation, and systemic inflammatory vascular disease. The proposed pathways contributing to liver injury during orthohantavirus infection are summarized in Figure 2, which illustrates the interplay between endothelial infection, cytokine release, sinusoidal injury, and hepatocellular stress.

### 3.3. Immunopathogenesis of Hepatic Involvement

The marked clinical heterogeneity observed in orthohantavirus infection suggests that disease severity depends not only on viral characteristics but also on the nature and intensity of host immune responses. Increasing evidence indicates that dysregulated antiviral immunity, rather than uncontrolled viral replication alone, plays a major role in the development of endothelial dysfunction and multiorgan injury, including hepatic involvement [1,3,31,32]. Understanding these immunopathogenetic mechanisms is essential for explaining why some patients develop hepatitis-like presentations while others do not.

Unlike many highly cytopathic viruses, orthohantaviruses can persist within endothelial cells while initially avoiding robust innate immune activation [1,30,31]. Experimental studies have shown that several hantavirus species interfere with interferon-mediated antiviral pathways, thereby facilitating early viral dissemination and modulating downstream inflammatory responses [30,31,32]. Klimaj and colleagues recently demonstrated that Seoul orthohantavirus can evade innate immune activation within reservoir endothelial cells, highlighting the importance of host-specific antiviral interactions in viral persistence and pathogenicity [30]. This evasion strategy may allow the virus to establish infection without triggering immediate immune clearance, creating a window during which endothelial dysfunction can develop progressively.

The host inflammatory response appears to represent a double-edged sword. While antiviral immunity is essential for viral clearance, excessive immune activation may contribute directly to vascular injury and tissue dysfunction. Elevated concentrations of pro-inflammatory cytokines, chemokines, and endothelial activation markers have consistently been associated with severe disease phenotypes [3,42,43,44]. In particular, IL-6 signaling has emerged as a potentially important mediator linking immune activation to endothelial barrier disruption [42]. In the liver, excessive cytokine exposure can amplify sinusoidal inflammation, recruit activated leukocytes, and contribute to hepatocellular stress even without direct viral infection of hepatocytes.

Innate immune dysregulation may also influence organ-specific manifestations. Altered interferon signaling, aberrant cytokine production, and excessive leukocyte activation can amplify endothelial permeability and inflammatory tissue damage across multiple organs, including the liver [31,32]. This mechanism may partly explain why some patients present with predominantly renal or pulmonary involvement, whereas others develop broader systemic manifestations characterized by hepatic abnormalities, gastrointestinal symptoms, or multiorgan dysfunction. Genetic polymorphisms in innate immune pathways may influence these patterns, although this remains an area of active investigation.

Host-specific immune responses likely contribute to the differing pathogenic profiles observed between Old World and New World hantaviruses. Experimental models suggest that viral species differ in cellular tropism, immune activation patterns, and interactions with endothelial and epithelial barriers [33,35]. Jeyachandran and colleagues recently demonstrated differential tissue tropisms between Old and New World hantaviruses, findings that may help explain variations in disease severity and organ involvement across geographic regions [35]. Whether these differences extend to hepatic tropism or the propensity to cause hepatitis-like presentations remains unknown and warrants further study.

Pericytes have also emerged as potential contributors to immune-mediated vascular injury. Beyond their structural role in microvascular stability, pericytes participate actively in inflammatory signaling and endothelial regulation. Infection of pericytes by Andes virus has been associated with enhanced endothelial permeability, suggesting that vascular dysfunction results from coordinated interactions between multiple cellular components of the microvascular environment [29]. In the liver, the sinusoidal microvasculature lacks classical pericytes but contains hepatic stellate cells, which share some functional characteristics. Whether hepatic stellate cells are susceptible to orthohantavirus infection or contribute to liver injury through similar mechanisms is an intriguing question for future research.

Another important aspect involves the balance between protective and pathogenic immune responses. Excessive activation of antiviral pathways may promote endothelial damage through release of inflammatory mediators, platelet activation, and coagulation abnormalities. Conversely, insufficient antiviral control may permit prolonged viral persistence and sustained inflammatory activation, leading to chronic or relapsing symptoms. This dynamic interaction may partly account for the unpredictable clinical course observed in some patients, including progression from mild febrile illness to severe systemic disease with hepatic involvement within a relatively short timeframe [3].

Importantly, many of these immunopathogenic mechanisms overlap with pathways implicated in other viral hemorrhagic and systemic inflammatory syndromes, including severe dengue, leptospirosis, and sepsis. The resulting clinical picture may therefore resemble severe dengue, leptospirosis, sepsis, or acute viral hepatitis accompanied by systemic inflammatory response and capillary leakage. Recognizing these overlapping mechanisms is essential for improving diagnostic suspicion and understanding the broader multisystem nature of orthohantavirus disease, particularly in patients presenting with fever, thrombocytopenia, and hypertransaminasemia.

## 4. Differential Diagnosis and Diagnostic Challenges

One of the major clinical challenges posed by orthohantavirus infection is its remarkable overlap with several infectious syndromes frequently encountered in tropical medicine, emergency care, hepatology, and internal medicine. Early manifestations are often nonspecific and may include fever, myalgia, headache, gastrointestinal symptoms, thrombocytopenia, elevated inflammatory markers, and abnormal liver enzymes, findings that can closely resemble a broad spectrum of viral, bacterial, and zoonotic diseases [2,3,36]. This diagnostic ambiguity is particularly pronounced when hepatic manifestations predominate, as the resulting clinical picture may be indistinguishable from acute viral hepatitis or other febrile hepatotropic syndromes. Understanding these overlaps and their clinical implications is essential for reducing misclassification and improving patient outcomes.

### 4.1. Overlap with Dengue, Leptospirosis, and Viral Hepatitis

The clinical overlap between orthohantavirus infection and other tropical and febrile illnesses is substantial and often bidirectional. In regions where dengue is endemic, orthohantavirus infection may initially be misclassified as arboviral disease because both conditions share features such as fever, capillary leakage, thrombocytopenia, hemoconcentration, and hepatic involvement [39]. Similarly, diagnostic overlap has been described with leptospirosis, another zoonotic infection characterized by febrile illness, renal dysfunction, thrombocytopenia, jaundice, and transaminase elevation [38]. Şahin and colleagues highlighted that clinical and routine laboratory findings alone are often insufficient to reliably distinguish hantavirus infection from leptospirosis during the early stages of disease, emphasizing the importance of epidemiological context and targeted microbiological testing [38].

The overlap becomes even more complex when hepatic manifestations predominate. In some patients, hypertransaminasemia may represent one of the earliest or most evident laboratory abnormalities, creating a clinical scenario highly suggestive of acute viral hepatitis [36,37]. This diagnostic ambiguity is particularly relevant in patients presenting with fever and elevated aminotransferases in the absence of overt renal or pulmonary involvement. Under these circumstances, clinicians may preferentially investigate hepatotropic viruses such as hepatitis A, B, C, or E, as well as Epstein--Barr virus and cytomegalovirus, while failing to consider orthohantavirus infection within the differential diagnosis. The consequence is a predictable diagnostic delay during which appropriate monitoring for capillary leakage, thrombocytopenia, or evolving renal dysfunction may be omitted.

Another complicating factor is the broad variability in disease severity. Mild or moderate cases may remain clinically indistinguishable from self-limited viral syndromes, whereas severe presentations can mimic septic shock, fulminant hepatitis, or systemic inflammatory disorders with multiorgan failure [3,48,50,51]. The increasing recognition of atypical phenotypes suggests that the traditional distinction between renal and cardiopulmonary syndromes may substantially underestimate the true multisystem spectrum of hantavirus-associated disease. In particular, patients with predominant hepatic involvement may not fit neatly into either classical category, further contributing to diagnostic confusion.

Imported infections and climate-driven ecological changes further complicate diagnostic recognition. As human mobility increases and rodent habitats evolve in response to climate change, clinicians practicing in non-endemic areas may encounter sporadic or unexpected cases without immediately considering hantavirus infection [48,50]. This issue may be particularly relevant in Europe, where underdiagnosis and limited awareness probably contribute to a significant underestimation of the true disease burden [41]. Autochthonous cases are increasingly recognized across the continent, yet many clinicians remain unfamiliar with the diverse presentations of orthohantavirus disease.

The possibility of person-to-person transmission in selected viral species also has important diagnostic implications. The Andes virus outbreak described in Argentina demonstrated that transmission chains may occur outside classical environmental exposure settings, thereby broadening the epidemiological scenarios in which clinicians should maintain suspicion [48]. In such contexts, the absence of direct rodent exposure should not automatically exclude the diagnosis. A family cluster of febrile illness with hepatitis-like features, for example, might be misattributed to a common viral infection when orthohantavirus transmission should be considered.

Overall, these diagnostic overlaps reinforce the need for a broader syndromic approach when evaluating patients with febrile illness associated with thrombocytopenia, endothelial dysfunction, renal abnormalities, or unexplained hepatic involvement. Orthohantavirus infection should be considered particularly in patients with compatible epidemiological exposures, atypical hepatitis presentations, or systemic inflammatory features not fully explained by more common infectious etiologies. The major differential diagnoses of hepatitis-like orthohantavirus presentations are summarized in Table 4, which provides a practical guide to distinguishing features and diagnostic considerations.

**Figure 3:**
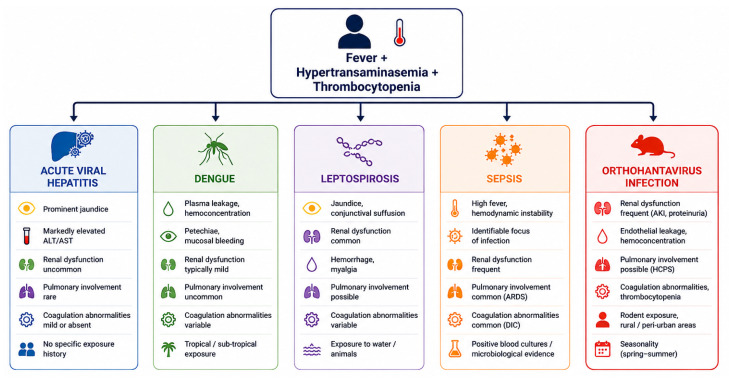
Differential diagnosis of orthohantavirus infection presenting with hepatitis-like syndrome. The figure provides a practical diagnostic algorithm for patients presenting with the triad of fever, hypertransaminasemia, and thrombocytopenia. Key discriminating features are outlined for five conditions that frequently enter the differential diagnosis: acute viral hepatitis, dengue, leptospirosis, sepsis, and orthohantavirus infection. For each condition, distinguishing clinical and laboratory characteristics are highlighted, including the presence or absence of renal dysfunction, pulmonary involvement, coagulation abnormalities, jaundice, plasma leakage, hemodynamic instability, and specific epidemiological clues such as rodent exposure or seasonality. This algorithm is intended to assist clinicians in recognizing orthohantavirus infection among other febrile hepatotropic syndromes, particularly when hepatic manifestations predominate and classical renal or pulmonary features are still evolving or absent.

The overlap between orthohantavirus infection and other febrile hepatotropic syndromes may significantly complicate early diagnosis, and a proposed diagnostic approach for hepatitis-like presentations potentially associated with orthohantavirus infection is shown in Figure 3.

### 4.2. Clinical Implications of Misclassification

Failure to recognize orthohantavirus infection may have significant clinical and public health consequences. Misclassification can delay appropriate supportive management, postpone monitoring for complications, and lead to incomplete epidemiological investigation. In patients initially labelled as having uncomplicated viral hepatitis or nonspecific febrile illness, the evolution toward renal dysfunction, capillary leakage, respiratory deterioration, or hemodynamic instability may therefore be underestimated during the early phases of disease [2,3,27]. This delay can be particularly dangerous because the transition from mild prodromal symptoms to severe systemic involvement may occur rapidly, sometimes within hours.

This issue is particularly relevant because no single clinical feature is pathognomonic during the initial presentation. In many patients, the first abnormalities are nonspecific laboratory findings such as thrombocytopenia, leukocytosis, elevated C-reactive protein, mild coagulation alterations, or transaminase elevation [45,46]. Consequently, diagnostic orientation is often driven by local epidemiology and clinician familiarity rather than by distinctive biological signatures. In non-endemic settings, orthohantavirus infection may therefore remain entirely absent from the initial diagnostic work-up, leading to a cascade of testing for more familiar conditions before the correct diagnosis is considered.

The underrecognition of hepatic involvement contributes further to this problem. Patients presenting predominantly with fever and hypertransaminasemia are frequently investigated for hepatitis A, B, C, and E, Epstein–Barr virus, cytomegalovirus, dengue, leptospirosis, drug-induced liver injury, or autoimmune hepatitis before zoonotic viral syndromes are considered [36,38]. Although this approach is clinically reasonable given the relative frequencies of these conditions, it may inadvertently delay hantavirus testing, especially when renal manifestations are absent or still evolving. In some cases, the diagnosis may never be pursued, contributing to persistent underreporting.

Importantly, several reports suggest that hepatic abnormalities may coexist with only subtle renal impairment during the early disease course [36,37]. This creates a potentially misleading clinical picture in which liver involvement appears disproportionate compared with other organ manifestations. A patient with fever, jaundice, marked transaminase elevation, and only a modest rise in creatinine may be correctly evaluated for viral hepatitis but incorrectly assumed not to have a zoonotic infection. The consequence is that orthohantavirus infection may remain clinically silent from an epidemiological perspective, contributing to persistent underdiagnosis and underreporting across many regions.

Diagnostic suspicion should therefore rely heavily on exposure history and contextual epidemiology. Occupational or environmental exposure to rodents, rural activities, military training, forestry work, farming, or exposure to contaminated dust may provide essential clues supporting the diagnosis [2,3]. However, exposure history may not always be evident, particularly in urban settings or imported cases. Urban residents may have had unrecognized exposure during travel, recreational activities, or even through peridomestic rodent contact. In Europe, changing ecological conditions and climate-associated shifts in rodent distribution may further modify traditional exposure patterns, making classical epidemiological assumptions progressively less reliable [40,41].

Another important implication concerns infection control and outbreak recognition. Although most orthohantaviruses are not associated with sustained human-to-human transmission, Andes virus represents a notable exception [48]. Delayed diagnosis in this setting may complicate contact tracing and epidemiological containment efforts during clusters of infection. During the Argentine outbreak, failure to recognize the index case as orthohantavirus infection allowed transmission chains to propagate before public health interventions were implemented. Awareness of atypical clinical presentations, including hepatitis-like forms, therefore has implications extending well beyond individual patient management.

From a hepatology perspective, recognizing orthohantavirus infection within the differential diagnosis of acute hepatitis-like syndromes may help avoid unnecessary invasive procedures or inappropriate therapeutic interventions. In most cases, liver injury appears secondary to systemic endothelial and inflammatory mechanisms rather than direct hepatocellular cytopathic destruction [1,28,29,42]. Consequently, management remains primarily supportive and focused on careful monitoring of systemic complications. Unnecessary liver biopsy, immunosuppressive therapy for suspected autoimmune hepatitis, or antiviral treatment for presumed viral hepatitis can be avoided when the correct diagnosis is established.

Overall, the available evidence suggests that orthohantavirus infection should no longer be viewed exclusively as a renal or pulmonary disease. Instead, clinicians should consider it a systemic endothelial syndrome with heterogeneous organ involvement, including clinically relevant hepatic manifestations that can dominate the initial presentation. Greater awareness among hepatologists, infectious disease specialists, emergency physicians, and intensivists may improve diagnostic recognition and facilitate earlier multidisciplinary management, ultimately reducing morbidity and preventing unnecessary interventions.

## 5. Biomarkers and Prognostic Implications

The clinical course of orthohantavirus infection is highly heterogeneous, ranging from mild self-limited disease to fulminant syndromes characterized by shock, respiratory failure, severe acute kidney injury, coagulopathy, and multiorgan dysfunction [3,50,51]. This wide variability in outcomes has stimulated growing interest in identifying biomarkers capable of predicting disease progression and supporting early risk stratification. Such tools are particularly valuable because the transition from nonspecific prodromal symptoms to life-threatening organ failure can occur rapidly, often within hours. In the context of hepatic involvement, biomarkers may also provide indirect insight into the mechanisms underlying hypertransaminasemia and help distinguish orthohantavirus-associated liver injury from other causes of acute hepatitis.

### 5.1. Biomarkers of Disease Severity

Among the most consistently reported abnormalities are hematological and inflammatory alterations reflecting endothelial injury and immune activation. Thrombocytopenia remains one of the hallmark laboratory findings and is frequently associated with disease severity, vascular leakage, and systemic inflammation [2,43,46]. The degree of platelet reduction often correlates with the intensity of capillary leakage and may serve as an early warning sign of progressing disease. In parallel, leukocyte abnormalities and elevated inflammatory indices may reflect the intensity of host immune dysregulation during acute infection, although these findings are less specific than thrombocytopenia.

Recent studies have highlighted the potential prognostic value of the neutrophil-to- lymphocyte ratio (NLR), a simple and widely available marker of systemic inflammation. Nusshag and colleagues demonstrated that an elevated NLR correlates with markers of severe disease during acute hantavirus infection, suggesting that dysregulated innate immune responses contribute substantially to clinical deterioration [45]. Because this ratio is rapidly obtainable from a routine complete blood count, it may represent a pragmatic tool for early bedside assessment, particularly in resource-limited settings where more sophisticated biomarkers are unavailable. An increasing NLR over serial measurements may signal worsening inflammation and impending clinical deterioration.

Coagulation abnormalities also appear closely linked to disease severity. Chen and colleagues reported that alterations in coagulation parameters, together with inflammatory and biochemical markers, correlate with prognosis in patients with hemorrhagic fever with renal syndrome [46]. These findings are biologically plausible given the central role of endothelial dysfunction and capillary leakage in hantavirus pathogenesis. The interaction between endothelial activation, platelet consumption, and inflammatory signaling likely contributes to the characteristic hemorrhagic manifestations observed in severe cases. Prolonged prothrombin time, elevated D-dimer, and fibrin degradation products may all signal progressive microvascular injury and increased risk of bleeding complications.

Additional biomarkers associated with endothelial injury have recently emerged as promising prognostic indicators. Wei and colleagues identified soluble thrombomodulin as a potential marker for risk stratification and outcome prediction in hemorrhagic fever with renal syndrome [47]. Thrombomodulin is a transmembrane protein expressed on the surface of endothelial cells; it is released into the circulation during endothelial damage. Elevated circulating levels of soluble thrombomodulin may therefore reflect the extent of vascular injury and microcirculatory dysfunction. Similarly, studies evaluating biomarkers in Puumala virus infection have demonstrated associations between markers of endothelial permeability, such as angiopoietin-2 and vascular endothelial growth factor, and clinical severity [30,43]. These endothelial-derived markers may prove particularly useful in identifying patients at risk of progressing to severe disease before overt organ failure develops.

Cytokine dysregulation represents another central component of severe orthohantavirus disease. Maleki and colleagues showed that IL-6 trans-signaling contributes to cytokine secretion and endothelial barrier dysfunction in hantavirus-infected cells and correlates with disease severity [42]. Elevated IL-6 levels have been consistently associated with more severe clinical phenotypes, including greater capillary leakage, more pronounced thrombocytopenia, and higher rates of multiorgan involvement. These findings reinforce the concept that severe manifestations are driven not only by viral replication itself but also by an exaggerated host inflammatory response capable of amplifying vascular permeability and tissue injury. Other pro-inflammatory cytokines, including TNF-α and IL-1β, have also been implicated, although IL-6 appears to be the most consistently associated with poor outcomes.

Interestingly, many of these biomarkers are not organ-specific and instead reflect a broader systemic endothelial syndrome. This may partly explain why patients can develop simultaneous renal, pulmonary, hepatic, and hematological abnormalities despite heterogeneous clinical phenotypes. A patient with marked elevation of soluble thrombomodulin and IL-6, for example, is likely to have more extensive endothelial dysfunction and consequently a higher risk of multiorgan involvement, including the liver. In the context of hepatic involvement, inflammatory and endothelial biomarkers may therefore provide indirect insight into the mechanisms underlying hypertransaminasemia, even when liver biopsy or dedicated hepatic imaging is not performed.

Although several candidate biomarkers appear promising, current evidence remains limited by heterogeneity in study design, viral species, geographic distribution, and disease definitions. What holds true for Puumala virus in Scandinavia may not fully apply to Hantaan virus in Asia or Andes virus in South America. Standardized validation across diverse cohorts will therefore be necessary before these markers can be routinely incorporated into clinical algorithms. Nevertheless, the increasing identification of prognostic indicators represents an important step toward earlier recognition of severe disease and more individualized patient monitoring. Several laboratory abnormalities have now been consistently associated with disease severity and adverse outcomes in both HFRS and HCPS, providing a foundation for future risk stratification approaches.

### 5.2. Prognostic Models and Risk Stratification

Beyond isolated biomarkers, increasing efforts have focused on the development of integrated prognostic models capable of identifying patients at higher risk of severe disease progression. This need is clinically relevant because deterioration in orthohantavirus infection may occur rapidly, often after an initially nonspecific prodromal phase characterized by fever, malaise, gastrointestinal symptoms, or mild laboratory abnormalities [2,3]. A patient who appears stable on admission may decompensate within hours, underscoring the value of tools that can identify those requiring closer monitoring or transfer to a higher level of care.

Recent studies have proposed multivariable approaches combining inflammatory, hematological, renal, and coagulation parameters to improve early risk assessment. Ma and colleagues developed the EASTAR model, a prognostic nomogram derived from a multicenter retrospective cohort of patients with hemorrhagic fever with renal syndrome [52]. The model incorporated routine clinical and laboratory variables, including age, platelet count, white blood cell count, creatinine level, and coagulation parameters, to predict severe disease progression and demonstrated encouraging discriminatory performance. Although external validation in independent cohorts remains necessary, this approach reflects the broader trend toward structured risk stratification in hantavirus-associated disease. The EASTAR model is particularly attractive because it relies on widely available laboratory tests, making it applicable even in resource-constrained settings.

Similarly, Wang and colleagues recently proposed a novel critical risk stratification scale for patients with Hantaan virus infection, integrating laboratory and clinical indicators associated with adverse outcomes [49]. Their scale incorporated variables such as shock, neurological involvement, bleeding manifestations, and laboratory markers of organ dysfunction. These efforts are particularly important in settings where access to advanced intensive care support may be limited and where early identification of high-risk patients could facilitate timely transfer to tertiary centers, thereby potentially reducing mortality.

Several studies suggest that markers reflecting endothelial dysfunction, inflammatory activation, and coagulation abnormalities are among the strongest predictors of severe outcomes [28,29,43,44,45,46,47]. This observation is coherent with the current understanding of hantavirus pathogenesis, in which vascular instability and dysregulated host responses represent central drivers of organ injury. Elevated inflammatory cytokines, thrombocytopenia, leukocyte alterations, and endothelial biomarkers such as soluble thrombomodulin may therefore collectively identify patients with a more aggressive systemic phenotype. The combination of multiple abnormal biomarkers likely confers greater prognostic value than any single marker alone.

Importantly, prognostic patterns may differ between Old World and New World hantaviruses, and risk stratification tools must account for these differences. While hemorrhagic fever with renal syndrome is classically associated with renal involvement and capillary leakage, New World hantaviruses frequently produce rapidly progressive cardiopulmonary syndromes characterized by respiratory failure and shock [3,51]. Tortosa and colleagues, in a recent systematic review and meta-analysis, identified several prognostic factors associated with mortality in New World hantavirus infections, emphasizing the importance of early hemodynamic instability and severe pulmonary involvement as predictors of poor outcome [51]. In contrast, for Old World hantaviruses, renal failure severity and coagulation abnormalities may be more prominent prognostic indicators.

The growing recognition of atypical and extrapulmonary manifestations further complicates prognostic evaluation. Patients presenting predominantly with hepatic abnormalities, gastrointestinal symptoms, or nonspecific inflammatory syndromes may initially appear clinically stable despite underlying endothelial dysfunction already being active. Under these circumstances, reliance exclusively on classical renal or pulmonary indicators could underestimate disease severity during the early stages of infection. A patient with fever, marked hypertransaminasemia, and mild thrombocytopenia but normal creatinine and oxygen saturation might be discharged home if evaluated only through a traditional lens, yet this same patient could evolve toward renal failure or pulmonary edema within days. Integrated prognostic models that incorporate inflammatory and endothelial biomarkers, regardless of the dominant organ involved, may help identify such patients earlier.

From a practical perspective, risk stratification tools may also help optimize resource allocation and determine the appropriate intensity of monitoring. Patients with severe thrombocytopenia (platelet count below 50,000 per microliter), rapidly increasing inflammatory markers (such as C-reactive protein or NLR), evidence of capillary leakage (hemoconcentration, hypoalbuminemia), coagulation abnormalities (prolonged prothrombin time, elevated D-dimer), or evolving organ dysfunction may benefit from closer hemodynamic surveillance and earlier multidisciplinary management involving infectious disease specialists, intensivists, nephrologists, and hepatologists. In contrast, patients with mild thrombocytopenia, normal renal function, and only modest transaminase elevation may be candidates for outpatient monitoring if adequate follow-up can be assured.

Despite recent advances, prognostic modeling in orthohantavirus infection remains an evolving field. Most available studies are retrospective, geographically heterogeneous, and frequently limited to specific viral species or regional outbreaks. The EASTAR model, for example, was developed primarily from patients with HFRS in China and may not directly generalize to Puumala virus infections in Europe or Andes virus infections in South America. Larger international cohorts and standardized clinical definitions will be necessary to improve generalizability and clarify whether existing models can be applied across different hantavirus syndromes. Prospective validation studies are urgently needed.

Nevertheless, the emergence of validated prognostic approaches represents an important development in the clinical management of orthohantavirus infection. As awareness of atypical disease manifestations, including hepatitis-like presentations, continues to increase, integrated models combining biomarkers, endothelial dysfunction parameters, and systemic inflammatory indicators may become increasingly valuable for recognizing severe disease before irreversible organ injury develops. Key biomarkers and prognostic indicators associated with severe disease are summarized in Table 3, which provides a practical overview of the most promising markers and their clinical significance.

## 6. Immunopathogenesis and Host–Virus Interactions

The pathogenesis of orthohantavirus infection is characterized by a complex interaction between viral tropism, endothelial dysfunction, and dysregulated host immune responses. Unlike many directly cytopathic viral infections, tissue injury in hantavirus disease appears to result predominantly from immune-mediated and vascular mechanisms rather than extensive direct cellular destruction [1,2,3]. This concept is particularly important for understanding the heterogeneous clinical spectrum of disease, including hepatic involvement, and for identifying potential therapeutic targets.

### 6.1. Endothelial Dysfunction and the Microvascular Unit

Endothelial cells represent the principal cellular target of orthohantaviruses. Viral infection of the vascular endothelium contributes to increased capillary permeability, plasma leakage, tissue edema, and multiorgan dysfunction without necessarily causing overt endothelial cell lysis [1,28]. The preservation of endothelial structural integrity despite profound functional dysregulation is considered one of the defining biological features of hantavirus infection. This unique characteristic distinguishes hantavirus disease from other viral hemorrhagic fevers, such as Ebola or severe dengue, where direct cytopathic effects are more prominent.

Experimental studies have progressively clarified several mechanisms involved in endothelial barrier disruption. Gavrilovskaya and colleagues demonstrated that hantaviruses sensitize endothelial cells to VEGF, thereby amplifying vascular permeability pathways [28]. Under normal conditions, VEGF plays a role in physiological angiogenesis and vascular homeostasis. However, in the context of hantavirus infection, infected endothelial cells become hyperresponsive to VEGF, leading to excessive permeability even at physiological concentrations of this growth factor. Additional evidence suggests that endothelial hyperpermeability may be further enhanced through activation of inflammatory signaling cascades involving Src family kinases and the TLR4/TRAF6 pathway [3]. These processes converge on the disruption of intercellular junctions, including VE-cadherin and tight junction proteins, resulting in paracellular leakage and the characteristic capillary leakage syndrome underlying renal, pulmonary, hepatic, and systemic manifestations.

Beyond endothelial cells, recent studies indicate that pericytes, specialized contractile cells that surround the endothelium in capillaries and venules, may also participate in disease pathogenesis. Pericytes play a crucial role in maintaining microvascular stability, regulating blood flow, and coordinating endothelial barrier function. Perez and colleagues demonstrated that Andes virus can infect pericytes and subsequently enhance endothelial permeability through paracrine interactions [29]. Infected pericytes release soluble mediators that act on adjacent endothelial cells, amplifying barrier dysfunction even when endothelial cells themselves are not directly infected. This finding broadens the current understanding of the microvascular unit involved in hantavirus disease and suggests that vascular dysfunction extends beyond endothelial cells alone. The concept of a “multi-cellular vascular unit”, comprising endothelial cells, pericytes, and basement membrane components, represents an important paradigm shift in understanding hantavirus pathogenesis and may help explain the variability in organ involvement among different patients.

### 6.2. Immune Dysregulation and Host–Virus Interactions

Innate immune responses play a central role in determining disease severity. Orthohantaviruses have evolved sophisticated mechanisms capable of modulating antiviral signaling pathways and interfering with host innate immunity [30,31,32]. Viral evasion strategies may delay effective antiviral responses while simultaneously promoting dysregulated inflammatory activation. For example, several hantavirus species have been shown to inhibit RIG-I-like receptor signaling, thereby reducing type I interferon production during the early stages of infection. This delayed interferon response allows viral replication to proceed unchecked, leading to a higher viral load and a more robust but poorly regulated inflammatory response once the immune system eventually activates. Species-specific differences in immune interactions may also partly explain the variability in clinical manifestations observed among different hantavirus species and hosts [32,33]. Rodent reservoir hosts typically sustain persistent, asymptomatic infection, whereas incidental human hosts frequently develop severe inflammatory disease, highlighting the importance of host-specific antiviral interactions in viral persistence and pathogenicity [30].

Recent investigations have highlighted the importance of cytokine-mediated endothelial injury. Elevated levels of proinflammatory cytokines, including interleukin-6 (IL-6), tumor necrosis factor-alpha (TNF-α), and other mediators of immune activation, appear closely associated with severe disease phenotypes [42]. Maleki and colleagues demonstrated that IL-6 trans-signaling, a pathway in which IL-6 complexes with soluble IL-6 receptor to activate cells that do not express the membrane-bound receptor, contributes directly to endothelial barrier dysfunction in infected cells and correlates with disease severity in HFRS patients [19]. This finding is particularly relevant because IL-6 trans-signaling is known to be pathogenic in several chronic inflammatory diseases, and its blockade has therapeutic potential. Other cytokines and chemokines, including CXCL10 (IP-10), CCL2 (MCP-1), and IL-8, recruit activated lymphocytes and monocytes to sites of infection, amplifying local inflammation and contributing to tissue injury.

At the same time, excessive immune activation may contribute to tissue injury independently of viral burden. Studies evaluating innate and adaptive immune responses suggest that exaggerated inflammatory signaling, activation of cytotoxic lymphocytes (particularly CD8+ T cells), and endothelial-immune interactions may collectively promote organ dysfunction [31,32,35]. In severe HFRS and HCPS, high numbers of activated CD8+ T cells are found in peripheral blood and in affected tissues, including the kidneys, lungs, and liver. These cells produce perforin and granzymes, which can damage endothelial cells and contribute to capillary leakage. The resulting syndrome resembles a systemic inflammatory endothelial disorder rather than a conventional organ-specific viral infection. This immunopathogenic framework explains why viral load does not always correlate with disease severity and why immunomodulatory therapies have been explored in severe hantavirus disease.

Host–virus interactions may also differ between Old World and New World hantaviruses. Experimental evidence indicates substantial variability in cellular tropism, immune modulation, and virulence profiles among distinct viral species [35]. These differences may partly account for the predominance of renal syndromes in some regions and cardiopulmonary manifestations in others. Importantly, however, overlapping mechanisms of endothelial dysfunction and immune dysregulation appear common to both clinical phenotypes.

The hepatic manifestations observed in some patients likely emerge within this broader framework of systemic vascular and immune injury. Rather than reflecting exclusive hepatotropism, liver abnormalities may result from a combination of endothelial leakage within the hepatic sinusoids, inflammatory cytokine exposure, microcirculatory dysfunction, and systemic immune activation [1,29,36,42]. The liver, with its highly specialized and permeable sinusoidal endothelium, is particularly vulnerable to these perturbations. This interpretation is consistent with the observation that hypertransaminasemia frequently occurs alongside evidence of multisystem endothelial involvement and does not follow a pattern typical of direct viral hepatitis.

Current evidence supports the concept that orthohantavirus infection should be viewed as a dynamic host–pathogen interaction involving endothelial dysfunction, immune dysregulation, and heterogeneous organ susceptibility. Continued investigation into these mechanisms may not only improve understanding of atypical presentations such as hepatitis-like disease, but also identify potential targets for future host-directed therapeutic strategies, including immunomodulatory agents that attenuate harmful inflammation without compromising viral clearance.

## 7. Public Health and Epidemiological Perspectives

The epidemiology of orthohantavirus infection is undergoing progressive transformation under the influence of ecological, climatic, and societal changes. Although traditionally considered geographically restricted zoonotic infections associated with specific rodent reservoirs, hantaviruses are increasingly recognized as dynamic emerging pathogens whose distribution and clinical relevance may evolve over time [3,40]. Approximately thirty thousand human cases are reported annually worldwide, although due to widespread underreporting, particularly of mild or atypical presentations, the true burden is likely substantially higher [53]. This shifting landscape has important implications for clinicians, public health authorities, and surveillance systems, particularly as atypical presentations, including hepatitis-like illness, become more frequently recognized.

### 7.1. Climate, Ecology, and the One Health Framework

Climate variability appears to play a particularly important role in shaping transmission dynamics. Environmental changes influencing rodent population density, food availability, habitat distribution, and human exposure patterns may substantially affect viral circulation. In a recent pan-European assessment, Guo and colleagues identified climate, biodiversity, and socio-economic factors as major determinants of human hantavirus risk across Europe [40]. Warmer winters and milder autumns, for example, have been associated with increased survival and reproduction of rodent reservoirs, leading to population outbreaks that precede human cases by several months.

A systematic review by Tagliapietra and colleagues further elucidated the ecological determinants driving orthohantavirus prevalence in small mammals across Europe, identifying five key focus areas that influence viral circulation: host characteristics (age, sex, reproductive maturity), population dynamics (abundance and density), abiotic factors (local and regional climate), habitat characteristics (vegetation cover, landscape changes, and masting patterns of deciduous trees), and interactions with other species including non-host rodents and predators [53]. Understanding these multifaceted drivers is essential for predicting human outbreak risk, as patterns of human cases do not consistently correspond with virus distribution within host populations, and peak prevalence in reservoir hosts does not necessarily coincide with peak human incidence [53]. The relationship between host population density and human cases is further complicated by factors such as winter survival rates, ecological conditions, and the challenges of forecasting host population trends across diverse European landscapes [53].

These findings reinforce the concept that hantavirus epidemiology should be interpreted within a broader One Health framework that integrates environmental, ecological, and human determinants [53,54]. Such an approach recognizes that human health cannot be separated from the health of animal populations and the ecosystems they inhabit. Hantavirus disease exemplifies the need for One Health principles, as it encompasses human patients and the animal reservoir, which may spread the disease without notice [54]. In Europe, the most prevalent orthohantavirus is Puumala virus (PUUV), which accounts for over 95% of reported human infections and causes nephropathia epidemica, a milder form of hemorrhagic fever with renal syndrome, with its main reservoir being the bank vole (*Myodes glareolus*) [55]. Other clinically relevant orthohantaviruses in Europe include Dobrava virus (DOBV), which can cause severe HFRS with up to 12% fatality and is carried by the yellow-necked mouse (*Apodemus flavicollis*), as well as Seoul virus (SEOV) associated with brown rats, and Tula virus (TULV) carried by common voles [53].

### 7.2. Land Use Change, Urbanization, and Human Exposure

Urbanization, land-use modifications, agricultural activities, deforestation, and expanding human encroachment into wildlife habitats may further increase opportunities for rodent–human interaction. As natural habitats are fragmented or destroyed, rodents often adapt to peri-urban and agricultural environments, bringing them into closer contact with human populations. Activities such as farming, forestry, military training, camping, and even cleaning rodent-infested buildings have all been associated with increased infection risk. Forestry workers, outdoor enthusiasts, and rural inhabitants are generally considered at higher risk due to their prolonged contact with potentially infected hosts and their habitat [53]. The impact of land use change on hantavirus disease risk has been particularly well documented in the Neotropics. A study analyzing 24 years of hantavirus cardiopulmonary syndrome transmission risk in Brazil found that amounts of native forest and sugarcane cultivation, combined with temperature, were the most important factors influencing disease risk, with sugarcane expansion likely promoting interactions between rural workers and infected rodents [56]. The study also identified that population at risk (rural workers) and rodent host diversity had a positive effect on disease risk, demonstrating the importance of understanding interactions between landscape change, rodent diversity, and hantavirus disease incidence [56]. These findings suggest that land use policy should consider disease risk as part of broader public health planning.

At the same time, global mobility and international travel create conditions in which imported or unexpected cases may increasingly be encountered outside historically endemic regions. A recent global analysis of orthohantavirus threats and regional trends highlighted that while China has demonstrated success in reducing infections through targeted public health interventions including vaccination programs, significant regional disparities persist worldwide [57]. Russia maintains stable infection rates, Nordic countries experience high incidence further exacerbated by climate change, South America shows rising case numbers, and Africa represents an emerging concern. The review identified multiple challenges to prevention and control, including the strong adaptability of rodent reservoirs, high rates of asymptomatic infection, weak primary healthcare and disease surveillance systems in many regions, diverse and evolving viral genotypes, low public awareness, and ecological changes that may exacerbate transmission. Consequently, clinicians practicing in areas traditionally considered low-risk may still face sporadic cases presenting with atypical or nonspecific clinical manifestations, and a negative history of travel to known endemic areas should not automatically exclude hantavirus infection from the differential diagnosis.

### 7.3. Underrecognition and Underreporting in Europe and Beyond

In Europe, awareness of hantavirus infection remains variable and likely insufficient in many clinical settings. Riccò and colleagues emphasized that the apparent rarity of hantavirus disease in Italy may partly reflect underrecognition and underreporting rather than true absence of infection [41]. This issue may be especially relevant for mild or atypical presentations that fail to fulfill classical definitions of hemorrhagic fever with renal syndrome or hantavirus cardiopulmonary syndrome. In countries where hantavirus testing is not routinely available or is reserved only for severe cases meeting strict clinical criteria, the true incidence of infection may be substantially underestimated. Seroprevalence studies in several European populations have revealed rates of prior infection significantly higher than the number of reported clinical cases, supporting the existence of a large iceberg of unrecognized or misdiagnosed illness.

The underdiagnosis of hepatic involvement probably contributes to this hidden epidemiological burden. Patients presenting primarily with febrile hepatitis-like syndromes may undergo extensive evaluation for hepatotropic viruses, including hepatitis A, B, C, E, Epstein–Barr virus, and cytomegalovirus, while zoonotic etiologies remain overlooked. As highlighted in earlier sections, orthohantavirus-associated hypertransaminasemia may occur even in the absence of overt renal or pulmonary manifestations [3,36,37]. Without targeted suspicion, these cases may never be microbiologically confirmed, and the opportunity for accurate epidemiological tracking is lost. This is particularly problematic because even mild or self-limited cases contribute to the overall burden of disease and may provide early warning signals of increased viral circulation in rodent reservoirs.

The heterogeneity of clinical phenotypes also complicates surveillance systems. Current case definitions in many regions remain strongly oriented toward classical renal or pulmonary syndromes, potentially overlooking patients with extrapulmonary manifestations or incomplete presentations. Broader recognition of endothelial and multisystem involvement may therefore have implications not only for clinical diagnosis but also for epidemiological surveillance strategies. Future surveillance strategies may benefit from integrating ecological monitoring, climate-based risk prediction, and broader syndromic diagnostic approaches that capture atypical presentations [53,56].

## 8. Conclusions

Orthohantavirus infection should no longer be viewed exclusively as a renal or cardiopulmonary disease. The accumulating body of evidence reviewed in this work indicates that hepatic involvement represents a relatively common, yet frequently underrecognized, component of the multisystem clinical spectrum of hantavirus disease. In a substantial subset of patients, liver abnormalities may dominate the initial presentation, closely mimicking acute viral hepatitis and thereby complicating early diagnosis. This diagnostic challenge is not merely academic; it has direct consequences for patient management, including delays in appropriate supportive care, unnecessary diagnostic procedures, and missed opportunities for early monitoring of potentially life-threatening complications such as capillary leakage, shock, and acute kidney injury.

The pathogenic mechanisms underlying hepatic injury in orthohantavirus infection differ fundamentally from those of classical hepatotropic viral hepatitis. Current evidence strongly supports a model in which liver abnormalities arise secondarily to systemic endothelial dysfunction, immune-mediated inflammation, and microvascular leakage, rather than from direct cytopathic infection of hepatocytes. The hepatic sinusoidal endothelium, with its unique fenestrated architecture and high permeability, appears particularly vulnerable to the vascular dysregulation that characterizes severe hantavirus disease. Within this framework, elevated aminotransferases may be best understood as a hepatic expression of a broader systemic inflammatory vascular syndrome, rather than as evidence of isolated liver disease. This conceptual shift has important implications for clinical management: interventions directed at supporting endothelial integrity, modulating excessive inflammation, and preventing organ failure are likely to be more beneficial than hepatocidal antiviral strategies.

From a diagnostic perspective, several practical considerations emerge. Clinicians evaluating patients with acute febrile illness, thrombocytopenia, and unexplained hypertransaminasemia, particularly in the absence of an obvious alternative diagnosis, should maintain a low threshold for considering orthohantavirus infection. Epidemiological clues such as rodent exposure, rural or occupational activities, and compatible seasonality or geographic location should be actively sought, although their absence should not exclude the diagnosis, especially in the setting of imported cases or emerging transmission patterns. The differential diagnosis should include dengue, leptospirosis, sepsis, drug-induced liver injury, and autoimmune hepatitis, but orthohantavirus infection deserves a place on this list, even in regions traditionally considered non-endemic. Routine laboratory markers such as thrombocytopenia, elevated neutrophil-to-lymphocyte ratio, and coagulation abnormalities may provide supportive evidence, while specific serological or molecular testing (IgM, IgG, RT-PCR) should be pursued when clinical suspicion is moderate to high.

The public health and epidemiological implications of underrecognizing orthohantavirus infection are substantial. The true burden of disease is almost certainly higher than reported figures suggest, due to a combination of mild or subclinical infections, atypical presentations that evade classical case definitions, and limited diagnostic capacity in many regions. Climate change, land-use modification, and increasing human–wildlife interface are likely to expand the geographic range and intensity of transmission, bringing orthohantaviruses into areas where clinicians have little familiarity with these infections. The recent documentation of person-to-person transmission for Andes virus further complicates the epidemiological landscape and underscores the need for rapid diagnostic recognition during outbreak situations. A broader, more flexible approach to surveillance, one that incorporates syndromic case definitions inclusive of hepatic presentations, integrates ecological and climate data, and leverages One Health principles, will be essential for tracking and responding to the evolving epidemiology of orthohantavirus disease.

Several important knowledge gaps remain and should guide future research efforts. First, prospective studies are needed to better define the true prevalence, spectrum, and natural history of hepatic involvement in orthohantavirus infection across different viral species and geographic settings. Such studies should systematically collect liver function tests, imaging data, and, where feasible, histopathological or non-invasive biomarkers of liver injury (e.g., transient elastography, serum fibrosis markers) to characterize the pattern and severity of hepatic damage. Second, the precise mechanisms linking endothelial dysfunction to hepatocellular injury remain incompletely understood; experimental models incorporating hepatic sinusoidal endothelium, hepatic stellate cells, and hepatocyte cultures could help elucidate whether direct or indirect mechanisms predominate. Third, the prognostic significance of hypertransaminasemia, whether it independently predicts worse outcomes or merely serves as a marker of systemic inflammation, should be clarified through multivariable analyses in large, well-characterized cohorts. Fourth, validation of existing prognostic models (such as EASTAR) and emerging biomarkers (such as soluble thrombomodulin and IL-6 trans-signaling) in diverse patient populations and across different orthohantavirus species is urgently needed before these tools can be widely adopted in clinical practice. Finally, therapeutic studies exploring immunomodulatory approaches, including blockade of IL-6 trans-signaling, modulation of endothelial permeability pathways, or targeted support of endothelial integrity, should consider hepatic outcomes as part of their endpoint assessments.

In conclusion, orthohantavirus infection is a systemic endothelial disorder with heterogeneous organ involvement, and the liver is more frequently affected than traditionally appreciated. Recognizing the hepatitis-like presentations of hantavirus disease is not a niche concern for specialists in zoonotic infections; it is a practical clinical skill with direct implications for diagnostic accuracy, patient safety, and public health surveillance. Increased awareness among hepatologists, infectious disease specialists, emergency physicians, and intensivists, coupled with improved diagnostic algorithms and prognostic tools, can reduce underdiagnosis, prevent unnecessary interventions, and improve outcomes for patients with this important and evolving group of infections.

## Figures and Tables

**Table 1:** Principal clinically relevant orthohantavirus species, their geographic distribution, rodent reservoirs, associated clinical syndromes, and approximate case fatality rates (CFRs).

Species	Geographic Distribution	Rodent Reservoir	Clinical Syndrome	Approximate CFR
Hantaan virus (HTNV)	China, Korea, Russia, Far East Asia [4]	Striped field mouse (*Apodemus agrarius*) [5]	HFRS (severe) [6]	1–15% [7]
Dobrava-Belgrade virus (DOBV)	Balkans, Central/ Eastern Europe [8]	Yellow-necked mouse (*Apodemus flavicollis*) [9]	HFRS (severe) [10]	Up to 12% [11]
Seoul virus (SEOV)	Worldwide (commensal rat distribution) [12]	Brown rat (*Rattus norvegicus*) [12]	HFRS (mild–moderate) [13]	<1% [14]
Puumala virus (PUUV)	Northern and Central Europe [15]	Bank vole (*Myodes glareolus*) [16]	Nephropathia epidemica (mild HFRS) [15]	<0.5% [17]
Tula virus (TULV)	Central Europe [18]	Common vole (*Microtus arvalis*) [19]	Subclinical/mild HFRS [20]	Rare [20]
Sin Nombre virus (SNV)	North America [21]	Deer mouse (*Peromyscus maniculatus*) [21]	HCPS (severe) [22]	~35% [22]
Andes virus (ANDV)	Southern South America [23]	Long-tailed pygmy rice rat (*Oligoryzomys longicaudatus*) [24]	HCPS (severe); person-to-person transmission documented [25]	~35–40% [26]

**Table 2:** Clinical spectrum of orthohantavirus infection beyond classical renal and pulmonary syndromes.

Clinical Domain	Main Manifestations	Proposed Mechanisms
Renal involvement	AKI, proteinuria, hematuria	Endothelial dysfunction, capillary leakage [2,3,27]
Pulmonary involvement	Pulmonary edema, HCPS, respiratory failure	Increased vascular permeability [3,51]
Hepatic involvement	Hypertransaminasemia, hepatitis-like presentation	Cytokine-mediated injury, endothelial dysfunction [3,36,37]
Hematological abnormalities	Thrombocytopenia, coagulopathy	Platelet consumption, endothelial activation [43,45,46]
Systemic inflammatory manifestations	Fever, shock, multiorgan dysfunction	Hyperinflammatory response [48,50]

**Table 3:** Summary of published clinical studies and case series reporting hepatic involvement during orthohantavirus infection.

Study	Year	Country/Setting	Design	N	Hantavirus Species	Elevated Transaminases	Clinical Pattern	Notes
Lledó et al. [37]	2003	Spain	Serological survey	1083 sera; subgroup with hepatitis	PUUV, DOBV	Reported in hepatitis subgroup	Febrile hepatitis-like illness; some with jaundice	First Spanish evidence of hepatic involvement
van Leeuwen et al. [36]	2022	International (review)	Narrative review	Multiple cohorts	Multiple species	Common; mild–moderate AST/ALT elevation	Exotic viral hepatitis; overlap with dengue, leptospirosis	Comprehensive review of hepatotropic exotic viruses
Şahin et al. [38]	2024	Turkey	Retrospective comparative	87 (hantavirus vs. leptospirosis)	PUUV/DOBV	Present in both groups; not discriminatory alone	Febrile illness with renal dysfunction and thrombocytopenia	Overlap with leptospirosis; laboratory findings insufficient alone
Ekanayake et al. [39]	2025	Sri Lanka	Prospective, tertiary hospital	210 suspected dengue	Not typed (probable hantavirus by serology)	Present in confirmed cases	Misclassified as dengue; thrombocytopenia + transaminase elevation	Highlights misclassification risk in dengue-endemic settings

**Table 4:** Differential diagnosis of orthohantavirus infection with hepatic involvement. Main infectious and non-infectious conditions that may mimic orthohantavirus-associated hepatitis-like presentations, highlighting overlapping clinical features and distinguishing clues. Diagnostic considerations for each condition include viral hepatitis serology (acute viral hepatitis), arboviral testing (dengue), serology/PCR (leptospirosis), blood cultures and procalcitonin (sepsis), autoantibodies and IgG (autoimmune hepatitis), and detailed medication history (drug-induced liver injury).

Clinical Condition	Shared Clinical Features	Distinguishing Clues
Acute viral hepatitis (A, B, C, E, EBV, CMV)	Fever, elevated AST/ALT	Thrombocytopenia less prominent in classic hepatitis
Dengue	Fever, thrombocytopenia, transaminase elevation	Epidemiological context, plasma leakage patterns, hemoconcentration
Leptospirosis	Fever, renal dysfunction, jaundice, thrombocytopenia	Exposure to contaminated water or animals, conjunctival suffusion
Sepsis/septic shock	Shock, multiorgan dysfunction, elevated inflammatory markers	Identifiable infectious source (e.g., pneumonia, urinary tract infection)
Autoimmune hepatitis	Hypertransaminasemia, fatigue	Autoimmune markers, chronicity, female predominance
Drug-induced liver injury	Elevated AST/ALT, jaundice	Temporal relationship with medication or toxin exposure

## Data Availability

No new data were created or analyzed in this study.

## References

[1] Noack D., Goeijenbier M., Reusken C.B., Koopmans M.P., Rockx B.H. (2020). Orthohantavirus pathogenesis and cell tropism. Front. Cell. Infect. Microbiol..

[2] Tariq M., Kim D.M. (2022). Hemorrhagic fever with renal syndrome: Literature review, epidemiology, clinical picture and pathogenesis. Infect. Chemother..

[3] Vial P.A., Ferrés M., Vial C., Klingström J., Ahlm C., López R., Le Corre N., Mertz G.J. (2023). Hantavirus in humans: A review of clinical aspects and management. Lancet Infect. Dis..

[4] Ata G., Wang H., Bai H., Yao X., Tao S. (2021). Edging on mutational bias, induced natural selection from host and natural reservoirs predominates codon usage evolution in hantaan virus. Front. Microbiol..

[5] Klein T.A., Park K., Rajoriya S., Kim H.C., Kim J., Lee S.H., Gu S.H., Sames W.J., O’Guinn M.L., Turell M.J. (2025). Longitudinal study of Orthohantavirus hantanense in *Apodemus agrarius* and disease risk assessment in the Republic of Korea during 2000–2019. Sci. Rep..

[6] National Library of Medicine (2024). Hemorrhagic Fever with Renal Syndrome MeSH Descriptor Data 2026.

[7] Afzal S., Ali L., Batool A., Afzal M., Kanwal N., Hassan M., Safdar M., Ahmad A., Yang J. (2023). Hantavirus: An overview and advancements in therapeutic approaches for infection. Front. Microbiol..

[8] Stamenković G., Nikolić V., Blagojević J., Bugarski-Stanojević V., Adnađević T., Stanojević M., Vujošević M. (2015). Genetic Analysis of Dobrava–Belgrade Virus from Western Serbia–A Newly Detected Focus in the Balkan Peninsula. Zoonoses Public Health.

[9] Leopardi S., Drzewnioková P., Baggieri M., Marchi A., Bucci P., Bregoli M., De Benedictis P., Gobbo F., Bellinati L., Citterio C. (2022). Identification of Dobrava-Belgrade virus in *Apodemus flavicollis* from north-eastern Italy during enhanced mortality. Viruses.

[10] Klempa B., Tkachenko E.A., Dzagurova T.K., Yunicheva Y.V., Morozov V.G., Okulova N.M., Slyusareva G.P., Smirnov A., Kruger D.H. (2008). Hemorrhagic fever with renal syndrome caused by 2 lineages of Dobrava hantavirus, Russia. Emerg. Infect. Dis..

[11] Hoornweg T.E., Zutt I., De Vries A., Maas M., Hoogerwerf M.N., Avšič-Županc T., Korva M., Reimerink J.H., Reusken C.B. (2020). Development of a comparative European orthohantavirus microneutralization assay with multi-species validation and evaluation in a human diagnostic cohort. Front. Cell. Infect. Microbiol..

[12] Ling J., Verner-Carlsson J., Eriksson P., Plyusnina A., Löhmus M., Järhult J.D., van de Goot F., Plyusnin A., Lundkvist Å., Sironen T. (2019). Genetic analyses of Seoul hantavirus genome recovered from rats (*Rattus norvegicus*) in the Netherlands unveils diverse routes of spread into Europe. J. Med. Virol..

[13] Avšič-Županc T., Saksida A., Korva M. (2019). Hantavirus infections. Clin. Microbiol. Infect..

[14] Reynes J.M., Carli D., Bour J.B., Boudjeltia S., Dewilde A., Gerbier G., Nussbaumer T., Jacomo V., Rapt M.P., Rollin P.E. (2017). Seoul virus infection in humans, France, 2014–2016. Emerg. Infect. Dis..

[15] Milhano N., Korslund L., Evander M., Ahlm C., Vainio K., Dudman S.G., Andreassen Å. (2017). Circulation and diagnostics of Puumala virus in Norway: Nephropatia epidemica incidence and rodent population dynamics. APMIS.

[16] Tscherne A., Guardado-Calvo P., Clark J.J., Krause R., Krammer F. (2025). Puumala orthohantavirus: Prevalence, biology, disease, animal models and recent advances in therapeutics development and structural biology. Front. Immunol..

[17] Ahlm C. (1997). Distribution of Puumala Virus in Sweden. Ph.D. Thesis.

[18] Saxenhofer M., Schmidt S., Ulrich R.G., Heckel G. (2019). Secondary contact between diverged host lineages entails ecological speciation in a European hantavirus. PLoS Biol..

[19] Erdin M., Smura T., Kalkan K.K., Cetintas O., Cogal M., Irmak S., Matur F., Polat C., Sironen T., Sozen M. (2024). Detection of divergent Orthohantavirus tulaense provides insight into wide host range and viral evolutionary patterns. npj Viruses.

[20] Hofmann J., Kramer S., Herrlinger K.R., Jeske K., Kuhns M., Weiss S., Ulrich R.G., Krüger D.H. (2021). Tula virus as causative agent of hantavirus disease in immunocompetent person, Germany. Emerg. Infect. Dis..

[21] Monroe M.C., Morzunov S.P., Johnson A.M., Bowen M.D., Artsob H., Yates T., Peters C., Rollin P.E., Ksiazek T.G., Nichol S.T. (1999). Genetic diversity and distribution of Peromyscus-borne hantaviruses in North America. Emerg. Infect. Dis..

[22] Goodfellow S.M., Nofchissey R.A., Arsnoe D., Ye C., Lee S., Park J., Kim W.K., Chandran K., Whitmer S.L., Klena J.D. (2024). Case of Human Orthohantavirus Infection, Michigan, USA, 2021. Emerg. Infect. Dis..

[23] Medina R.A., Torres-Perez F., Galeno H., Navarrete M., Vial P.A., Palma R.E., Ferres M., Cook J.A., Hjelle B. (2009). Ecology, genetic diversity, and phylogeographic structure of Andes virus in humans and rodents in Chile. J. Virol..

[24] Torres-Pérez F., Ferrada N., Astudillo R., Ferrés M., Vial P.A., Marquet P.A., Parra A., Mertz G.J., Palma R.E. (2025). Hantavirus infections and small mammal diversity in Chile: No differences between protected and unprotected areas highlight the need for public health strategies. PLoS Negl. Trop. Dis..

[25] Ferrés M., Martínez-Valdebenito C., Angulo J., Henríquez C., Vera-Otárola J., Vergara M.J., Pérez J., Fernández J., Sotomayor V., Valdés M.F. (2020). Mother-to-child transmission of Andes virus through breast milk, Chile. Emerg. Infect. Dis..

[26] Riesle-Sbarbaro S.A., Kirchoff N., Hansen-Kant K., Stern A., Kurth A., Prescott J.B. (2023). Human-to-human transmission of Andes virus modeled in Syrian Hamsters. Emerg. Infect. Dis..

[27] Koehler F.C., Di Cristanziano V., Späth M.R., Hoyer-Allo K.J.R., Wanken M., Müller R.U., Burst V. (2022). The kidney in hantavirus infection—Epidemiology, virology, pathophysiology, clinical presentation, diagnosis and management. Clin. Kidney J..

[28] Gavrilovskaya I.N., Gorbunova E.E., Mackow N.A., Mackow E.R. (2008). Hantaviruses direct endothelial cell permeability by sensitizing cells to the vascular permeability factor VEGF, while angiopoietin 1 and sphingosine 1-phosphate inhibit hantavirus-directed permeability. J. Virol..

[29] Perez R.D., Gorbonova E.E., Mackow E.R. (2021). Novel infection of pericytes by Andes virus enhances endothelial cell permeability. Virus Res..

[30] Klimaj S.D., LaPointe A., Martinez K., Acosta E.H., Kell A.M. (2024). Seoul orthohantavirus evades innate immune activation by reservoir endothelial cells. PLoS Pathog..

[31] LaPointe A., Gale M., Kell A.M. (2023). Orthohantavirus replication in the context of innate immunity. Viruses.

[32] Kell A.M. (2022). Innate immunity to orthohantaviruses: Could divergent immune interactions explain host-specific disease outcomes?. J. Mol. Biol..

[33] Noack D., van den Hout M.C., Embregts C.W., van IJcken W.F., Koopmans M.P., Rockx B. (2024). Species-specific responses during Seoul orthohantavirus infection in human and rat lung microvascular endothelial cells. PLoS Negl. Trop. Dis..

[34] Dieterle M.E., Solà-Riera C., Ye C., Goodfellow S.M., Mittler E., Kasikci E., Bradfute S.B., Klingström J., Jangra R.K., Chandran K. (2021). Genetic depletion studies inform receptor usage by virulent hantaviruses in human endothelial cells. eLife.

[35] Jeyachandran A.V., Irudayam J.I., Dubey S., Chakravarty N., Daskou M., Zaiss A., Garcia G., Konda B., Shah A., Venkatraman A. (2025). Differential tropisms of old and new world hantaviruses influence virulence and developing host-directed antiviral candidates. PLoS Pathog..

[36] van Leeuwen L.P., de Jong W., Doornekamp L., van Gorp E.C., Wismans P.J., Goeijenbier M. (2022). Exotic viral hepatitis: A review on epidemiology, pathogenesis, and treatment. J. Hepatol..

[37] Lledó L., Klingström J., Gegúndez M.I., Plyusnina A., Vapalahti O., Saz J.V., Beltrán M., Sjölander K.B., Vaheri A., Plyusnin A. (2003). Hantavirus infections in Spain: Analysis of sera from the general population and from patients with pneumonia, renal disease and hepatitis. J. Clin. Virol..

[38] Şahin A.M., Çetin S., Şenel İ., Erdem-Çakır T., Aydın E., Yetkin M.A. (2024). The role of clinical and laboratory finding in the differential diagnosis of hantavirus and leptospirosis infections. J. Vector Borne Dis..

[39] Ekanayake E., Govinna M., Wakkumbura S., Samarajeewa Y., Arachchige N., Weerathunga A., Rajamanthri L., Ranawaka G., Pattiyakumbura T., Dasanayake D. (2025). Detection of probable hantavirus infections in clinically suspected dengue patients in a tertiary care hospital in Sri Lanka. BMC Infect. Dis..

[40] Guo J., Semenza J.C., Ecke F., Rizzoli A., Dagostin F., Ulrich R.G., Sjödin H., Treskova M., Rocklöv J. (2026). A pan-European assessment of multi-sector drivers of human hantavirus risk: Climate, biodiversity, and socio-economic factors as key determinants. Environ. Res..

[41] Riccò M., Peruzzi S., Ranzieri S., Balzarini F., Valente M., Marchesi F., Bragazzi N.L. (2021). Hantavirus infections in Italy: Not reported doesn’t mean inexistent. Acta Bio Med. Atenei Parm..

[42] Maleki K.T., Niemetz L., Christ W., Wigren Byström J., Thunberg T., Ahlm C., Klingström J. (2025). IL-6 trans-signaling mediates cytokine secretion and barrier dysfunction in hantavirus-infected cells and correlates to severity in HFRS. PLoS Pathog..

[43] Outinen T.K., Mäkelä S., Pörsti I., Vaheri A., Mustonen J. (2021). Severity biomarkers in Puumala hantavirus infection. Viruses.

[44] Zhao H.D., Sun J.J., Liu H.L. (2023). Potential clinical biomarkers in monitoring the severity of Hantaan virus infection. Cytokine.

[45] Nusshag C., Gruber G., Zeier M., Krautkrämer E. (2024). Neutrophil-to-lymphocyte ratio is elevated in acute hantavirus infection and correlates with markers of disease severity. J. Med. Virol..

[46] Chen W.j., Du H., Hu H.f., Lian J.q., Jiang H., Li J., Chen Y.p., Zhang Y., Wang P.z. (2024). Levels of peripheral blood routine, biochemical and coagulation parameters in patients with hemorrhagic fever with renal syndrome and their relationship with prognosis: An observational cohort study. BMC Infect. Dis..

[47] Wei M., Xiao Z., Cao J., Du X., Li M., Zhang R., Yang X., Wu S., Fan C., Zhang J. (2026). The role of soluble thrombomodulin (sTM) in risk stratification of hemorrhagic fever with renal syndrome and prognostic assessment. PLoS Negl. Trop. Dis..

[48] Martínez V.P., Di Paola N., Alonso D.O., Pérez-Sautu U., Bellomo C.M., Iglesias A.A., Coelho R.M., López B., Periolo N., Larson P.A. (2020). “Super-spreaders” and person-to-person transmission of Andes virus in Argentina. N. Engl. J. Med..

[49] Wang S., Wang F., Ma Y., Zhuang R., Zhang Y., Zhang C., Tang K., Wei Y., Zuo J., Xu X. (2025). Development and validation of a novel critical risk stratification scale for HFRS patients with Hantaan virus infection. Int. J. Infect. Dis..

[50] Lu W., Kuang L., Hu Y., Shi J., Li Q., Tian W. (2024). Epidemiological and clinical characteristics of death from hemorrhagic fever with renal syndrome: A meta-analysis. Front. Microbiol..

[51] Tortosa F., Ragusa M.A., Neumann I., Perre F., Guaresti G., Donato M., Izcovich A. (2026). Prognostic factors for mortality in patients infected with New World hantaviruses: A systematic review and meta-analysis. BMJ Open.

[52] Ma K., Wu T., Guo W., Wang J., Ming Q., Zhu J., Wang H., Chen G., Wang X., Yan W. (2025). Clinical characteristics and a novel prediction nomogram (EASTAR) for patients with hemorrhagic fever with renal syndrome: A multicenter retrospective study. Trop. Med. Infect. Dis..

[53] Fabbri D., Mirolo M., Tagliapietra V., Ludlow M., Osterhaus A., Beraldo P. (2025). Ecological determinants driving orthohantavirus prevalence in small mammals of Europe: A systematic review. One Health Outlook.

[54] Samples O.M., Arowolo J. (2025). Hantavirus (*Hantavirus* spp.). The One Health Model as Applied to Zoonotic Diseases.

[55] Razzauti M., Castel G., Cosson J.F. (2021). Impact of Landscape on Host–Parasite Genetic Diversity and Distribution Using the Puumala orthohantavirus–Bank Vole System. Microorganisms.

[56] Muylaert R.L., Sabino-Santos G., Prist P.R., Oshima J.E., Niebuhr B.B., Sobral-Souza T., Oliveira S.V.d., Bovendorp R.S., Marshall J.C., Hayman D.T. (2019). Spatiotemporal dynamics of hantavirus cardiopulmonary syndrome transmission risk in Brazil. Viruses.

[57] Song J.N., Chen D., Wang L.M., Jiang H. (2024). Global threats and regional trends: Navigating the complex landscape of human Orthohantavirus infections. Infect. Dis. Immun..

